# Prevalence, Awareness, Treatment, Control of Hypertension, and Availability of Hypertension Services for Patients Living With Human Immunodeficiency Virus (HIV) in Sub-Saharan Africa (SSA): A Systematic Review and Meta-analysis

**DOI:** 10.7759/cureus.37422

**Published:** 2023-04-11

**Authors:** Kimera Isaac Derick, Zahid Khan

**Affiliations:** 1 Research, Makerere University Joint AIDS Program, Kampala, UGA; 2 Acute Medicine, Mid and South Essex NHS Foundation Trust, Southend on Sea, GBR; 3 Cardiology, Bart’s Heart Centre UK, London, GBR; 4 Cardiology and General Medicine, Barking, Havering and Redbridge University Hospitals NHS Trust, London, GBR; 5 Cardiology, Royal Free Hospital, London, GBR

**Keywords:** systematic reviews and meta-analyses, world health organisation, sub-saharan africa, antihypertensive medications, acquired immune deficiency syndrome (aids), combination antiretroviral therapy, hypertension and therapy, cardio vascular disease, hiv diseases, hiv aids

## Abstract

Sub-Saharan Africa (SSA) is faced with a dual burden of hypertension and human immunodeficiency virus (HIV). In this review we sought to determine the prevalence, awareness, and control of hypertension among persons living with HIV (PLHIV), and the availability of hypertension services at the HIV care points in SSA. We searched the PubMed, Embase, Scopus, Cochrane library, Global index Medicus, African Journal online, and WHO Institutional Repository for Information Sharing (IRIS) for studies on the epidemiology of hypertension, and hypertension services for PLHIV in SSA. Twenty-six articles were identified for the review, with 150,886 participants; weighted mean of age 37.5 years and female proportion of 62.6%. The pooled prevalence was 19.6% (95% confidence interval [CI], 16.6%, 22.5%); hypertension awareness was 28.4% (95% CI, 15.5%, 41.3%), and hypertension control was 13.4% (95% CI, 4.7%, 22.1%). HIV-related factors like CD4 count, viremia, and antiretroviral therapy regimen were not consistently associated with prevalent hypertension. However, high body mass index (BMI) above 25 kg/m^2^ [odds ratio: 1.64, 95% CI (1.26, 2.02)] and age above 45 years [odds ratio: 1.44, 95% CI (1.08, 1.79)] were associated with prevalent hypertension. Even when PLHIV on ART were more likely to be screened for hypertension and monitored, there was infrequent screening and treatment of hypertension in most HIV clinics. Most studies recommended integrating of HIV and hypertension services. We report a high prevalence of hypertension in a relatively young population of PLHIV with suboptimal screening, treatment, and control of hypertension. We recommend strategies to integrate HIV and hypertension services.

## Introduction and background

Globally, Sub-Saharan Africa (SSA) accounts for 70% of all persons living with HIV (PLHIV) bearing the blunt of the human immunodeficiency virus (HIV) epidemic [1-2]. Notably, due to the ongoing rapid epidemiological transition, SSA has a high prevalence of hypertension and thus is faced with a dual burden of hypertension and HIV [3].

The PLHIV with hypertension are at higher risk of adverse cardiovascular disease (CVD) events compared to PLHIV without hypertension [4]. HIV infection is a recognized CVD risk factor, in that PLHIV are twice as likely to suffer a CVD and four times more likely to suffer a myocardial infarction [5-6]. The HIV infection is associated with 2.2 times more risk of CVD for PLHIV on ART and 1.6 times for ART naïve persons [6-7]. In addition, traditional CVD risk factors like dyslipidemia and hypertension contributed to a greater attributable risk for myocardial infarction at 41% and 43%, respectively, than HIV-related factors, underscoring the importance of early screening and diagnosis of CVD risk factor in PLHIV [8]. However, individual observational studies have revealed differing levels of hypertension prevalence, awareness, and control among PLHIV across SSA [9-10]. Furthermore, studies have shown that globally, the prevalence, awareness, and control of hypertension varies by region [11]. The World Health Organization (WHO) recommends routine integration of screening and management of hypertension among PLHIV [12-13]. However, there has been inconsistent implementation of this recommendation and PLHIV have continued to receive suboptimal hypertension care [14-16].

Over 80% of premature non-communicable diseases (NCDs) mortality occurs in low and middle income countries, with CVD causes contributing about two-third of these deaths, translating to 17.9 million deaths annually [17]. Globally, Sub-Saharan Africa is still the only region where infectious diseases are still the leading cause of mortality [18]. However, SSA is experiencing an epidemiological transition with NCDs progressively contributing more to the overall disease burden than infectious diseases [19]. It is estimated that by 2030 NCDs are set to contribute to a higher mortality than infectious, maternal, and neonatal causes of mortality combined in SSA [20]. If not addressed earlier, this will cost heavily on the already constrained and poorly resourced health systems, with negative impact on socioeconomic development.

More so in Africa, uncontrolled hypertension is a common denominator in most adverse cardiovascular and cerebrovascular events such as stroke, myocardial infarction, heart failure, and chronic kidney disease [21]. Hypertension ranked highest among the 20 contributors of disability-adjusted life-years in Sub Saharan Africa [22]. Hypertension is regarded as the strongest risk factor for stroke, and it is estimated that improved hypertension control would in turn prevent 30% of the strokes, 25% of the myocardial infarction, and 23% of the chronic kidney diseases [23-24]. Surveys in the general population indicate a large burden of low awareness, untreated and poor control of hypertension in Sub-Saharan Africa [25-26]. This makes hypertension control a public health priority especially for resource limited settings with a number of competing priorities where a small investment will yield high socioeconomic returns in preventing morbidity and mortality due to adverse CVD events [27]. 

While there is increased CVD risk due to HIV infection alone, the sub-optimal levels of hypertension screening and poor access to essential hypertension medicines in HIV clinics in SSA may disproportionately contribute to the poor CVD disease outcomes among PLHIV [28]. These gaps in service delivery could be addressed through health system strengthening and optimizing care if they are well understood. The current form and the extent of hypertension care offered to PLHIV in SSA is unknown [14]. Most health systems in SSA are disease centered and parallel with little documented overlap between HIV and hypertension services [29]. The vertical (disease-centered) health systems in SSA are reported to negatively affect hypertension care among PLHIV [9, 15, 30]. Mitambo et al. (2017) reported low levels of hypertension awareness among PLHIV, and that routine hypertension screening is uncommon in most HIV clinics [31].

There exists a critical knowledge gap in literature that this review has tried to address. First, the WHO has recommended integrated hypertension and HIV care since early 2014, however, the extent to which these recommendations have been implemented was unknown. Second, this review aimed to understand the kind of hypertension services offered to PLHIV and the existing barriers that may contribute to the sub-optimal levels of hypertension treatment and control in SSA. Data on the available hypertension services for PLHIV will inform appropriate resource distribution, and development of context appropriate strategies, to address the burden of hypertension in the aging PLHIV population.

A clear understanding of the prevalence of hypertension among PLHIV and the capacity of health systems to address the growing burden, will inform planning, contextualized implementation of health programs, and drawing informed policies. This systematic review of literature assessed the prevalence, awareness, treatment initiation, control, and the kind of hypertension services offered to PLHIV in SSA.

## Review

Aims

The aims of this review were to assess the prevalence, awareness, and control of hypertension among persons living with HIV in SSA and to establish the management offered to PLHIV with hypertension in SSA.

Ethical consideration

We received ethical approval from the University of South Wales research ethics committee. Participants’ informed consent was not sought as we used secondary data to answer the study question without interacting with participants or with data with participant’s identifiers.

Methodology

This was a systematic review and meta-analysis of original studies that reported on the prevalence, awareness, treatment, and control of hypertension among PLHIV in SSA. To understand the current prevalence, awareness, treatment, and control of hypertension among PLHIV, we systematically searched PubMed, Embase, Scopus, Cochrane library, Global index Medicus, African Journal online, and WHO IRIS for studies on the epidemiology of hypertension among PLHIV in SSA. In addition, we also searched Google Scholar for articles. We filtered for articles published in English from January 1, 2000 to July 31, 2022 among humans. We created a database and imported all the studies obtained from the literature search to EndNote® (Clarivate, Berkeley, CA) software [32], and used the same to remove duplicates. The investigator screened all articles by titles and abstracts for publications that were relevant to the study question. After, full text articles of the relevant studies were obtained that met the pre-defined inclusion criteria for data extraction.

Inclusion criteria

Original studies conducted in SSA among persons diagnosed with HIV reporting on the prevalence of hypertension with or without reporting on the awareness, treatment and control, and regardless of the anti-retroviral therapy initiation status.

Studies with participants aged 18 years and above living in SSA.

Studies that screened the entire study population, or random sampling of the defined population, with more than 70% response rate.

Defined criteria for clinic/office blood pressure measurement, hypertension diagnosis (≥140/90 mmHg), and device used.

Exclusion criteria

The exclusion criteria were case reports, case series, letters to editors and qualitative research, studies that did not include persons living with HIV, studies that did not report of the required outcomes, studies not in English language, and studies with participants less than 18 years old.

Data extraction

**Figure 1:**
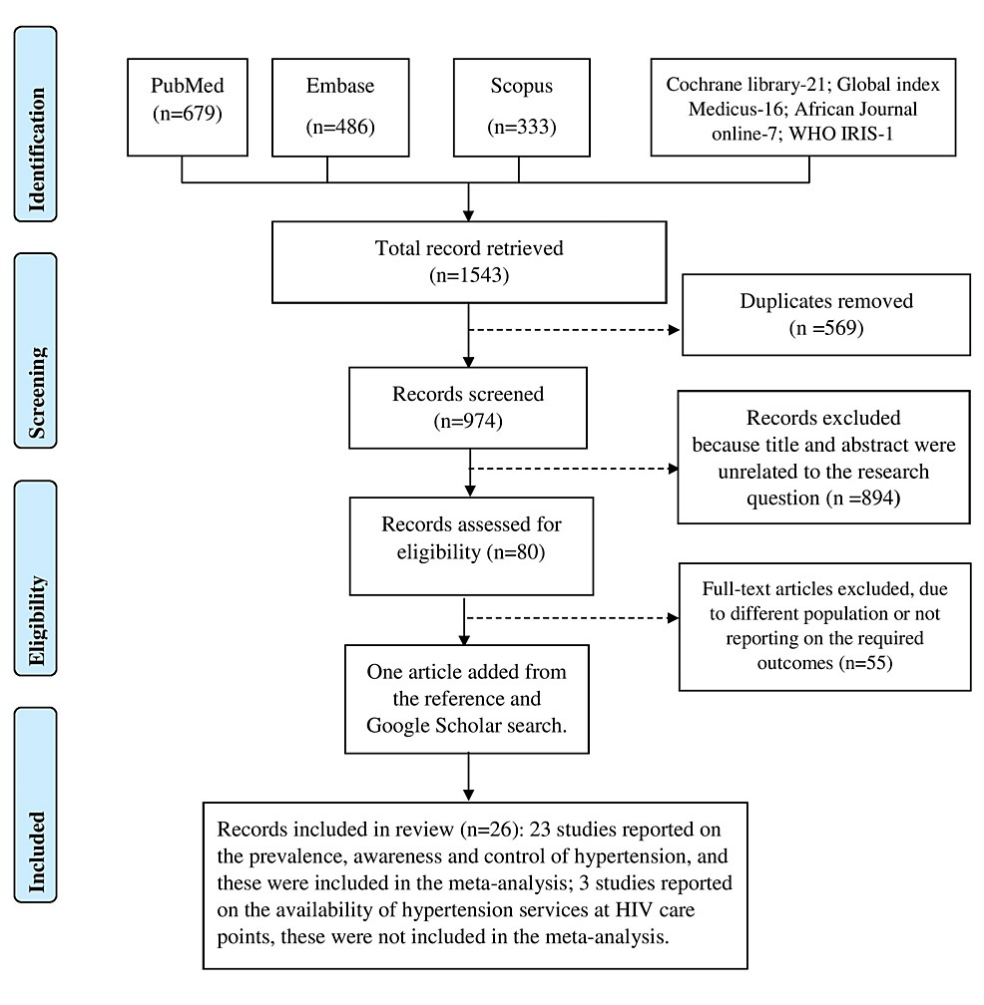
PRISMA chart. PRISMA, preferred reporting items for systematic reviews and meta-analyses

After obtaining full text articles that meet the inclusion criteria, we extracted data on: study author, publication year, year of data collection, country, study design, setting (urban versus rural), age limit and average age, female proportions, criteria used for blood pressure measurement and hypertension diagnosis. The extracted data were captured in a Microsoft Excel® (Microsoft, One Microsoft Way Redmond, Washington) data abstraction form. The investigator took effort to contact the corresponding authors of the selected relevant article in the case of missing or ambiguous data. All review data were stored on the investigator’s personal laptop and backup on Google cloud. We used the preferred reporting items for systematic reviews and meta-analyses (PRISMA) to develop the systematic review protocol. As per the protocol, we used free text and MESH terms for the literature search and obtained the following: total articles - 1543 (PubMed-679, Embase-486, Scopus-333, Cochrane library-21, Global index Medicus-16, African Journal online-7, WHO IRIS-1). The progression from the database search to the selection of articles that we included in the review is detailed in the PRISMA flow diagram (Figure 1).

The primary study outcomes were the prevalence, awareness, treatment, and control of hypertension among persons living with HIV in SSA. The prevalence was determined as the proportion of PLHIV screened and diagnosed with hypertension. The awareness, treatment initiation, and level of hypertension control were determined as a proportion of those diagnosed with hypertension. The secondary outcome was to understand the availability of routine hypertension services at HIV service points in SSA. We used the National Institute for Health Quality Assessment Tool for Observational Cohort and Cross-Sectional Studies, to grade the quality of the studies rating the overall score for each study as good, fair, or poor based on 14-item parameters [33]. We considered a score of < 5 as poor, 5-10 as fair, and > 10 as good. Most studies scored > 10, with the average quality assessment score being 11/14 (Appendix).

Most studies reported non-standardized prevalence of hypertension; therefore, we used standard errors to calculated confidence intervals of the variables. To obtain an overall summary estimate of the prevalence across studies, we pooled the study-specific estimates using a random-effects meta-analysis model. We assessed study heterogeneity using the I2 statistics, with a value of I2 = 0%-25% considered as low heterogeneity, moderate I2= 26%-50% as moderate, and I2>50% as high heterogeneity. We explored sources of heterogeneity by comparing hypertension prevalence between subgroups defined by setting (urban versus rural), year of publication, mean age, and for studies with extreme prevalence. In addition, we compared the pooled prevalence obtained from using the random-effects meta-analysis model compared with the fixed effects meta-analysis model. We assessed the presence of publication bias using funnel plots and compared the pooled prevalence between larger and smaller studies. All analyses were performed using Review Manager Software (Cochrane, London, UK) [34]. The different mean ages, differing prevalence of CVD risk factors such as BMI, physical activity and diet among the study participants, the inconsistent criteria for hypertension diagnosis (frequency and intervals), and the varying types of blood pressure machines used, contributed to study heterogeneity.

Results

The size of participants in the studies ranged from 332 to 80560 participants, with a total of 150,886 participants. The mean age of participants ranged from 34 to 44 years, with a weighted mean age of 37.5 years. The female proportion ranged from 51.7% to 78.6%, with a weighted proportion of 62.6%. Ten studies were conducted in rural settings, 11 in urban settings while two were conducted in both urban and rural settings. Of the 23 studies, 11 (44%) were conducted in Eastern Africa and nine (36%) from Southern Africa. South African nations are relatively more urbanized, ethnically and socially diverse compared to East African nations, which could have contributed to study heterogeneity. Nine (39%) were cross-sectional studies and five (23%) were retrospective cohort study designs. Three studies, one from Tanzania, one from South Africa and one from Malawi investigated availability and readiness of HIV clinics to provide hypertension care to persons living with HIV, but these were not included in the meta-analysis.

Most studies indicated that there were parallel HIV and hypertension services in most health facilities, requiring patients to attend to these clinics at different times. There was poor access to hypertension medicines within the HIV clinics, but some health facilities had these medicines available at the outpatient department. In general, few studies reported on hypertension treatment initiation and control among PLHIV. The characteristics of the 23 studies included in the meta-analysis are summarized in Table 1.

**Table 1:** Characteristics of 23 studies that reported on hypertension prevalence among persons living with HIV. HIV, human immunodeficiency virus

Author and year	Country and setting	Study design	Sample size	Female proportion	Median age	Number screened	Proportion diagnosed with hypertension	Proportion aware of hypertension	Proportion on hypertension treatment	Proportion with controlled hypertension
Kwarisiima et al. (2019) [9]	Rural Uganda	Prospective Cohort	2,071			2,071	199 (10.0%)		89 (44.7%)	
Dzudie et al. (2021) [10]	Urban Cameroon	Cross sectional	9,839	6,513 (66.2%)	42	9,839	2,351 (23.9%)		28 (1.2%)	6 (0.2%)
Muddu et al. (2019) [15]	Urban Uganda	Retrospective	1,649	975 (59.1%)	37.6 ± 11.2	1,649	218 (13.2%)		181 (83.0%)	53 (24.3%)
Mutemwa et al. (2018) [29]	Rural South Africa	Cross sectional	827	642 (77.7%)	38.4 ± 9.0	827	341 (41.2%)	140 (41.0%)	106 (31.0%)	83 (24.3%)
Bauer et al. (2017) [35]	Urban Zambia	Prospective Cohort	896	467 (52.1%)	34 (29-40)	883 (98.5%)	98 (10.9%)	35 (35.7%)	14 (21.2%)	
Bloomfield et al. (2011) [36]	Rural Kenya	Retrospective	12,194	7,901 (64.8%)		12,194	1,064 (8.7%)			
Brennan et al. (2018) [37]	Urban South Africa	Prospective Cohort	80,560	47,239 (60.8%)	37 (31-44)	77,696 (96.4%)	17,126 (22.0%)		1,952 (24.0%)	
Chiwandire et al. (2021) [38]	Urban South Africa	Cross sectional	4,484	2,964 (4484)		4,484	641 (14.3%)			
Divala et al. (2016) [39]	Urban & Rural Malawi	Cross sectional	952	683 (71.7%)	43	952	226 (23.7%)	60 (26.5%)	29 (12.8%)	
Ekrikpo et al. (2018) [40]	Urban Nigeria	Cross sectional	4,925		34.3 ± 9.9	4,925	1,306 (26.5%)	17 (1.3%)		
Fiseha et al. (2019) [41]	Rural Ethiopia	Cross sectional	408	273 (66.9%)	37 ± 10.3	408	121 (29.7%)	31 (25.6%)	31 (25.6%)	
Gonah et al. (2020) [42]	Urban Zimbabwe	Cross sectional	17,784	11,035 (62.0%)		17,784	3,468 (19.5%)	1,238 (35.7%)		
Hoffman et al. (2021) [43]	Urban Malawi	Prospective Cohort	671	347 (51.7%)	44 (39-52)	671	255 (38.0%)	158 (23.5%)	158 (23.5%)	30 (11.8%)
Kalyesubula et al. (2016) [44]	Urban Uganda	Retrospective	1,996	1,309 (65.6%)		1,996	418 (20.9%)		96 (23%)	
Kansiime et al. (2019) [45]	Urban Uganda	Cross sectional	387	256 (66.1)	42	387	48 (12.4%)			
Kwarisiima et al. (2016) [46]	Rural Uganda	Retrospective	3,545	2,304 (65%)		3,545	390 (11.0%)	78 (20%)	55 (14%)	25 (6.4%)
Lubega et al. (2021) [47]	Urban and Rural Uganda	Retrospective	2,026	1,501 (74.1%)		2,026	192 (9.5%)		140 (72.9%)	
Lukas et al. (2021) [48]	Rural Ethiopia	Cross sectional	382	206 (53.9%)	35 (29-43)	382	42 (11.0%)			
Masika et al. (2017) [49]	Urban Kenya	Retrospective	3,333	2,316 (72.4)	39.7	3,197	246 (7.7%)		28 (11.4%)	
Manavalan et al. (2020) [50]	Rural Tanzania	Cross sectional	806	436 (78.6%)		555	105 (18.9%)	32 (29.6%)	10 (9.5%)	0 (0%)
Mathebula et al. (2020) [51]	Rural South Africa	Cross sectional	332	239 (72%)			115 (34.6%)			
Mutede et al. (2015) [52]	Rural Zimbabwe	Cross sectional	393	222 (56.6%)		393	137 (34.9%)	28 (20.6%)		
Sander et al. (2015) [53]	Rural Uganda	Prospective Cohort	426	302 (71.0%)		402	34 (8.0%)	18 (52.9%)	15 (44.1%)	

Hypertension screening, awareness, diagnosis, treatment initiation, and control

In Zambia, blood pressure was measured regularly at each clinic visit and documented in patient files, suggesting the feasibility of routine screening of hypertension in HIV care settings [35]. Similar studies elsewhere reported routine screening of hypertension in HIV clinics [45, 53]. However, this was inconsistent in some settings which reported that about 34.2% of the study participants had not been screened for hypertension despite their multiple interactions with the health system [15, 52]. PLHIV older than 50 years were more likely to be screened for hypertension compared to those less than 30 years (odds ratio, OR 2.37 95% CI: 1.71, 3.29) [15].

Kwarisiima et al. (2019) reported challenges with linkage of patients diagnosed with hypertension to NCD care following community screening outreaches and over one-third of the patients diagnosed had stage 2 hypertension of ≥ 160/100 mmHg at the time of screening, thus suggesting that the burden of undiagnosed hypertension was severe [9]. Bauer et al. reported that 62.9% of the patients screened and diagnosed with hypertension became aware of their elevated blood pressure status at the ART clinic, while 22.9% were diagnosed at a parallel outpatient department clinic and 14.3% at NCD clinic at a referral hospital [35]. In addition, they reported that in Zambia, the diagnosis of hypertension was primarily communicated to patients by nurses (60.0%) and less commonly by a physician/medical officer (graduate) (25.7%) or a clinical officer (undergraduate or diploma holder) (14.3%) [35]. And Sander et al. (2015) reported that only 4.2% of patients that were screened and diagnosed with hypertension had the diagnosis documented in the patient file by the health worker [53].

In a prospective cohort, of the 8,125 incident hypertension cases, 24.0% (n = 1,952) received medical treatment for hypertension at the same clinic and of these, 32.6% were treated within 3 months, 9.2% within 3-6 months, 15.0% (n = 294) within 6-12 months, and 43.1% at more than 12 months after hypertension diagnosis. Of the untreated participants with sufficient follow-up, 53.9% (n=4,849) still had elevated blood pressure at 6 months after their initial hypertension diagnosis. Over 60% of patients with incident hypertension still had high blood pressure after five months following treatment initiation on hypertension medicines, suggesting that either the medication was not effective or was not adhered to appropriately [37]. HIV viral suppression did not predict hypertension control (adjusted OR 1.1; 95% CI: 0.8-1.4) [46].

Incidence of hypertension

Brennan et al. reported an incident rate of 5.4 per 100 person-years (95% CI, 5.3, 5.6), with 8,125 (13.4%) incident hypertension cases diagnosed at a median of 13 months [interquartile range, IQR: 9-230] following ART initiation [37]. Also individuals with incident hypertension were slightly older (46 versus 42 years, p < 0.001), and had a higher BMI (>25 kg/m2) proportion (25.3% versus 22.3%, p < 0.001) without significant gender differences [43].

Predictors of hypertension were noted to be ART. Sander et al. reported that there was no significant difference in hypertension prevalence among PLHIV on ART versus those not on ART (8.2% for those on ART versus 7.8% for ART naive, p = 0.87) [53]. Patients initiated on Nevirapine were more likely to develop hypertension compared to those initiated on Efavirenz (hazard ratio, 1.27 95% CI: 1.13, 1.43) by Brennan et al. [37] while Lukas et al. [48] reported a similar association but differing odds ratios (OR) 4.61, (CI: 2.52, 8.3) for Efavirenz and (OR 2.36 CI: 1.7, 5.8) for Nevirapine. Patients initiated on Zidovudine had a 40% increased hazard of developing hypertension compared to those initiated on Tenofovir [37]. Treatment with protease inhibitors was not associated with increased risk of hypertension even after 540 days on treatment [36]. Duration on ART at 2 years was associated with hypertension (OR 1.15 CI: 1.07, 1.24), while ART use of more than 5 years was associated with a higher increased risk of hypertension (OR 2.57, CI: 1.24, 5.21) [10].

Another risk factor was CD4+ count clinical status and HIV suppression. There were differing results on the association of CD4 count with hypertension, with few studies reporting no association of hypertension with CD4 count after adjusting for confounders like age, BMI, and pre-ART CD4 count [29, 35, 49, 53]. However, Bloomfield et al. reported that among men aged 16 -35 years with CD4 counts >350/µL were more likely to have elevated systolic blood pressures compared to those with CD4 <200/µL (p=0.05), but this observation was not seen with men >45 years. The same trend was also not observed with diastolic blood pressure or among women [36]. In contrast, Brennan et al. found that patients with low CD4 < 50/µL count at ART initiation were at increased risk of incident hypertension compare to those with CD4 count of > 350/µL (hazard ratio, 1.25 CI: 1.03, 1.50) [37]. However, individuals initiating ART with poorer health status that is, WHO staging III/IV, and hemoglobin <10 g/dL had lower prevalent hypertension at ART initiation (risk ratio, 0.84 CI: 0.80, 0.87).

 Pre-hypertension was another risk factor and patients with pre-hypertension were twice as likely to experience incident hypertension (hazard ratio, 2.05 CI: 1.92, 2.19) underscoring the need to monitor these patients closely [37]. A BMI >25 kg/m2 among PLHIV was associated with greater risk of hypertension compared to those with a normal BMI (Table 2). One standard deviation in the BMI of 0.175 kg/m2 (95% CI: 0.142, 0.208) resulted in a standard deviation increase in the systolic blood pressure of 0.25 mmHg (95% CI: 0.218, 0.285) and a corresponding standard deviation increase in the diastolic blood pressure of 0.21 mmHg (95% CI: 0.196, 0.222) [37]. In contrast, no association was observed between BMI and hypertension control for those on hypertension treatment [43].

**Table 2:** Odds of hypertension with BMI in PLHIV. BMI, body mass index; PLHVIV, persons living with human immunodeficiency virus; CI, confidence interval; OR, odds ratio

Study	Country	Control BMI (kg/m^2^)	Comparison BMI (kg/m^2^)	OR	95% CI
Bauer et al. (2017) [35]	Zambia	< 25	≥ 25	4.07	1.94, 8.53
Bloomfield et al. (2011) [36]	Kenya	< 25	≥ 25	1.80	1.50, 2.16
Brennan et al. (2018) [37]	South Africa	< 25	≥ 30	1.70	1.60, 1.81
Divala et al. (2016) [39]	Malawi	< 25	≥ 25	3.67	1.56, 8.64
Ekrikpo et al. (2018) [40]	Nigeria	< 25	≥ 25	1.06	1.03, 1.08
Kalyesubula et al. (2016) [44]	Uganda	18-24	≥ 30	2.04	1.43, 2.89
Lubega et al. (2021) [47]	Uganda	< 25	≥ 25	2.10	1.43, 3.11
Masika et al. (2017) [49]	Kenya	< 25	≥ 25	2.20	1.50, 3.40
Mutede et al. (2015) [52]	Zimbabwe	< 25	≥ 25	2.18	1.40, 3.80
Sander et al. (2014) [53]	Uganda	< 25	≥ 25	1.15	1.05, 1.26

In almost all studies, age was a consistent predictor of hypertension as summarized in Table 3. Sander et al. reported (OR 1.08, 95% CI: 1.04, 1.12) with each additional year. Compared to patients < 30 years, there was an increased risk of hypertension for patients aged 40-49 (hazard ratio 1.49, 95% CI: 1.42, 1.54) and for those >50 (hazard ratio 2.00 CI: 1.91, 2.11) by Brennan et al. [37]. In women, age unlike BMI, was the stronger predictor of hypertension (OR 2.21, 95% CI 1.95-2.50) versus BMI (OR 1.72 95% CI: 1.40, 2.11). However, in men, BMI was a stronger predictor of hypertension (OR 2.41 95% CI: 1.88, 3.09) compared to age (OR 1.62 95% CI: 1.40, 1.87) [36].

**Table 3:** Odds of hypertension with age in PLHIV. PLHIV, persons living with human immunodeficiency virus; OR, odds ratio; CI, confidence interval

Study	Country	OR	CI
Dzudie et al. (2021) [10]	Cameroon	1.28	1.25, 1.32
Bauer et al. (2017) [35]	Zambia	1.50	1.20, 1.93
Bloomfield et al. (2011) [36]	Kenya	2.21	1.95, 2.50
Ekrikpo et al. (2018) [40]	Nigeria	1.04	1.03, 1.05
Gonah et al. (2020) [42]	Zimbabwe	2.5	1.42, 3.22
Kalyesubula et al. (2016) [44]	Uganda	3.12	2.00, 4.85
Kansiime et al. (2019) [45]	Uganda	3.17	1.87, 5.41
Lubega et al. (2021) [47]	Uganda	2.7	1.47, 4.85
Sander et al. (2014) [53]	Uganda	1.08	1.04, 1.12

Availability of medicines for hypertension

In Zambia, medicines for hypertension were accessible to patients at no cost, usually at outpatient pharmacies of the health facilities housing the HIV clinics, and clinicians in ART clinics could prescribe these drugs [35].

In Uganda medicines for hypertension were usually procured with funds assigned to each health facility by the government under the ministry of health, and when these medicines were out of stock, the patients were asked to buy out of pocket from private pharmacies [15]. In comparison, hypertension medicines were readily available at ART facilities in South Africa [29]. Table 4 summarizes the commonly prescribed hypertension medicines in the studies [29].

**Table 4:** Commonly prescribed hypertension medicines in Sub-Saharan Africa. CCB, calcium channel blocker; ACEI, angiotensin converting enzyme inhibitor; ARB, angiotensin receptor blocker; K+, potassium; Aprinox, bendroflumethiazide

Study	Country	CCB	Beta blockers	Loop diuretics	ACEI	ARB	Thiazide diuretics	K+ Sparing diuretics	Others
Kwarisiima et al. (2019) [9]	Uganda	33% (Nifedipine); 1% (Amlodipine	13% (Atenolol)	2% (Furosemide)	9% (Captopril)	-	54% (Aprinox)	-	-
Bauer et al. (2017) [35]	Zambia	28.6% (Nifedipine)	-	21.4% (Furosemide)	-	-	-	-	50% unspecific
Hoffman et al. (2021) [43]	Malawi	17.8% (Nifedipine, Amlodipine)	8.9% (Propranolol, Atenolol)	-	8.9% (Enalapril, Captopril, Telmisartan, Losartan)	64.6% (Hydrochlorothiazide, Chlorthalidone)	-	-	
Sander et al. (2014) [53]	Uganda	37.2% (Nifedipine)	39.7% (Atenolol and Propranolol)	13.2% (Furosemide)	0.3% (Captopril)		-	9.2% (Aprinox)	0.3% (Spironolactone)	

Kwarisiima et al, (2019) reported increased frequent clinic visits due to drug stock outs, which were not clinically indicated, as one of the barriers to hypertension control [9]. The absence of simplified treatment protocols contributed to variability in the prescribed hypertension medicines [15].

Access to hypertension services

In South Africa, PLHIV on ART were more likely to be screened for hypertension (OR 1.27, 95% CI, 1.04, 1.55) , have awareness about their hypertension diagnosis (OR 1.52, 95% CI: 1.12 to 2.05), were initiated on treatment for hypertension (OR 1.63, 95% CI: 1.21 to 2.19) and to receive counseling on lifestyle modification (OR 1.57, 95% CI: 1.11 to 2.22), compared to HIV negative participants or HIV positive individuals not on ART [53-54].

Challenges with documentation of hypertension care and treatment

Sander et al. (2015) reported quality gaps in training on screening, treatment, documentation, monitoring, and follow-up of hypertension [53]. Another study also demonstrated that data on anti-hypertensive medicine use was not well documented in ART clinics [35]. Similarly, poor documentation of blood pressure measurements and the prescribed anti-hypertensive medication in the patients’ files was found which may have contributed to underestimation estimation of prevalent hypertension [37]. Only 4.2% of patients that were screened and diagnosed with hypertension had the diagnosis documented in the patient file by the health worker [53].

Patients’ HIV and hypertension data were collected and stored separately in different databases at all HIV clinics. Data of 29.3% of PLHIV already diagnosed with hypertension did not reflect at the ART clinic while receiving care at a separate NCDs clinics, thus HIV clinics were unable to quantify the HIV and hypertension comorbidity [42].

Availability of services and resources for hypertension care in HIV clinics

Two studies assessed the availability services, and preparedness of HIV facilities to manage hypertension comorbidity [55-56]. The authors assessed the availability and training for staff; availability of treatment protocol; resources for screening, diagnosis and treatment of hypertension in PLHIV using the ECHO International Health standards for essential clinic equipment [57].

Of the 14 HIV clinics assessed, 43% were actively screening and treating patients with HIV and hypertension comorbidity; 21% had a protocol for hypertension treatment; 36% had standard blood pressure cuffs; urine dipsticks (78%), blood glucose (88%), and lipid panel (57%); 21% had a healthcare worker with training in hypertension management, and all facilities provided some form of education on lifestyle modification on tobacco cessation and weight control. All had stadiometers; 93% had adult weighing scales; 64% had tape measures, and 57% had patient examination beds. In comparison, 64.6% of lower health facilities and 56.3% of hospitals met the standards of availability of equipment for screening and management of hypertension. However, the authors did not report on availability of hypertension medicines, except simvastatin that was available in only 14% of the surveyed clinics. In health facilities with medicines for hypertension, the medicines were available at the outpatient dispensing points located within the general health clinics or hospitals that house the HIV clinics however, their staff and activities were usually separate. The health provider-to-patient ratio ranged from 1:23 to 1:26 [55].

**Figure 2:**
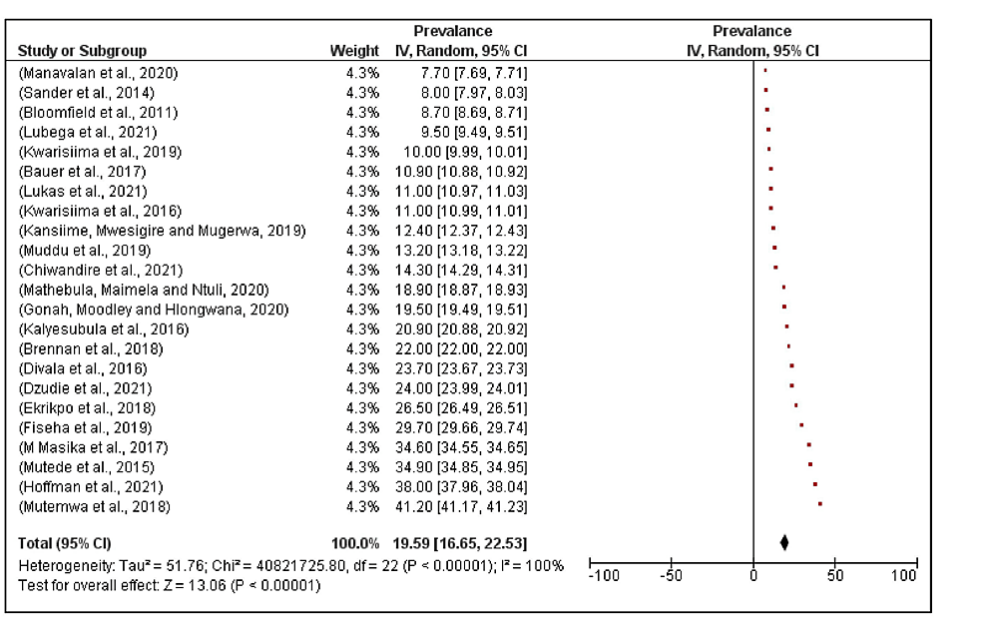
Prevalence of hypertension across 23 studies in Sub-Saharan Africa. The red boxes represent the effect estimates (prevalence) provided as a percentage. The diamond is the pooled effect estimate at 95% confidence interval (CI). [10, 15, 29, 35-53]

In a similar survey in Malawi, care and treatment of hypertension was predominantly by hospitals. Notably, 60% of hospitals had least one clinician and one nurse trained in NCD care on top of the formal training, while only 5% of lower health centers had a clinician and a nurse with extra NCD training. On equipment for hypertension screening, 100% of hospitals and 80% of health centers had at least one functional blood pressure machine. Only one of five hospitals routinely screened for hypertension in patients on ART and no health centers routinely screened for hypertension among PLHIV. However, health centers were more likely to provide integrated hypertension and HIV care once one was diagnosed hypertension. At least 48% (12/25) health centers provided integrated care during the same consultation compared to none of the hospitals. On the other hand, 60% (3/5) hospitals could provide patients with ART and hypertension treatment on the same day but patients had to see different clinicians [56]. Regarding availability of hypertension medicines, 100% of hospitals and 92% of health centers had uninterrupted supply of hydrochlorothiazide (first-line medical treatment), and 80% of hospitals and 96% of health centers had stock out of second line treatment for hypertension in the previous 6 months. All facilities reported that they were able to refer patients for emergency hypertension care when necessary but only but 50% had an ambulance, the alternative was public transport [56]. Overall, the prevalence of hypertension ranged from 7.7% in the study in Kenya with mean age of participants equal to 39.7 years [49] to 41.2% in South Africa with mean age of participants equal to 38.4 years [29]. The median prevalence was 18.9%, and the random effects model pooled prevalence across the 23 studies was 19.6% (95% CI, 16.6%, 22.5%; Figure 2).

**Figure 3:**
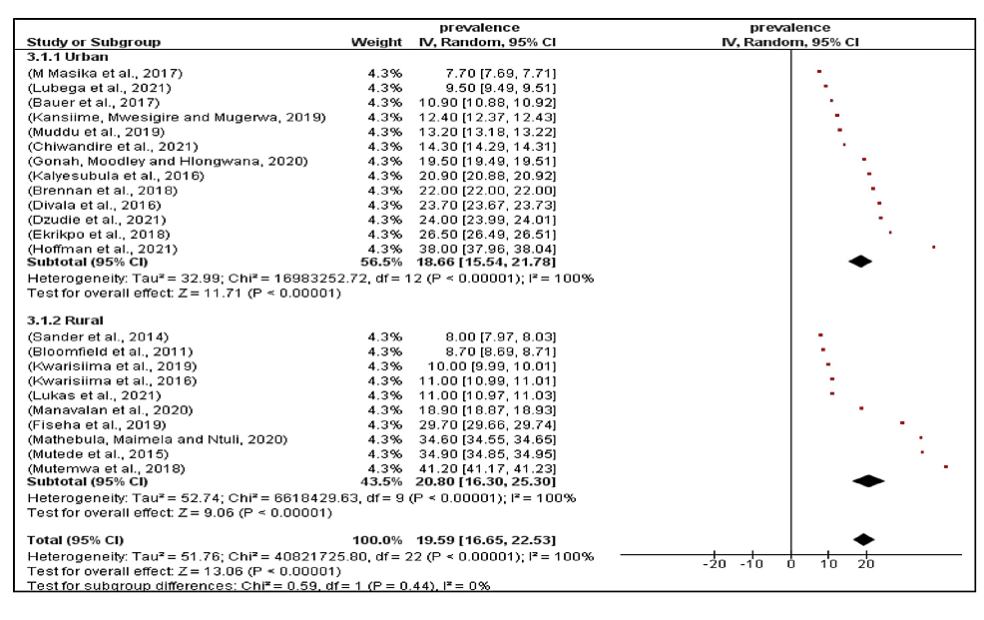
A sub-group analysis of the prevalence of hypertension among PLHIV living in urban versus rural settings in sub-Saharan Africa. The red boxes represent the effect estimates (prevalence) is provided as a percentage. The diamond is for the pooled effect estimate at 95% CI. PLHIV, persons living with HIV [9-10, 15, 29, 37-44, 46-48, 50-53]

There was no significant difference in the random effects model pooled prevalence between studies conducted in urban settings 18.7%, (95% CI, 15.4%, 21.8%) compared to those conducted in rural settings 20.8% (95% CI, 16.3%, 25.3%) (p=0.44) (Figures 2-3).

**Figure 4:**
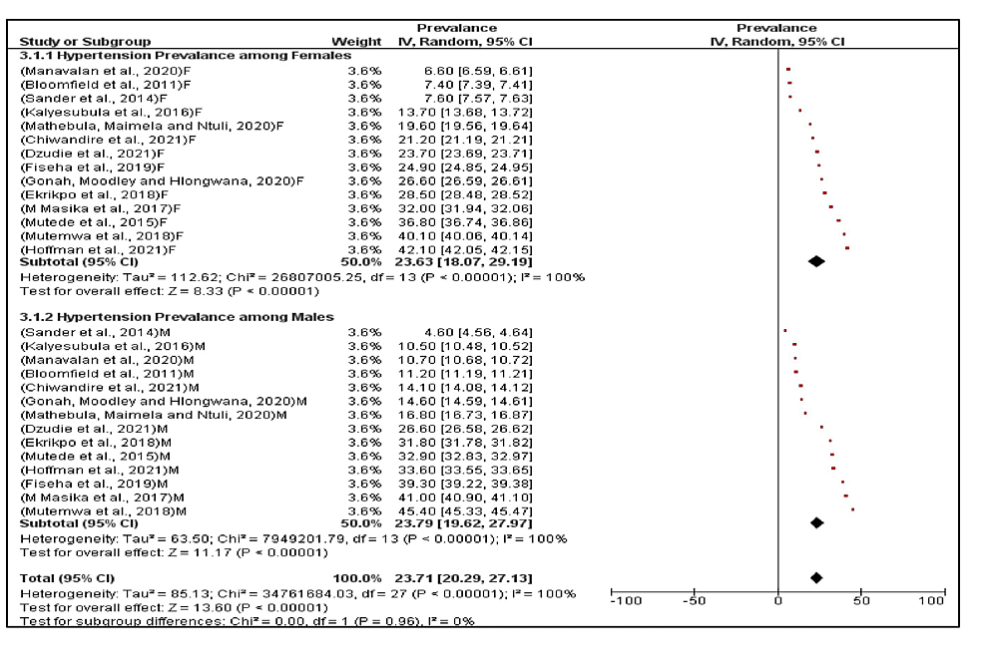
A sub-group analysis of the prevalence of hypertension among female versus male PLHIV in sub-Saharan Africa. Persons living with human immunodeficiency virus (PLHIV) [10, 29, 36, 38, 40-44, 50-53]

There was no significant difference in the random effects model pooled prevalence between females 23.6% (95% CI, 18.1%, 29.2%) and males 23.8% (95% CI, 19.6%, 28.0%) (p=0.96) (Figure 4).

**Figure 5:**
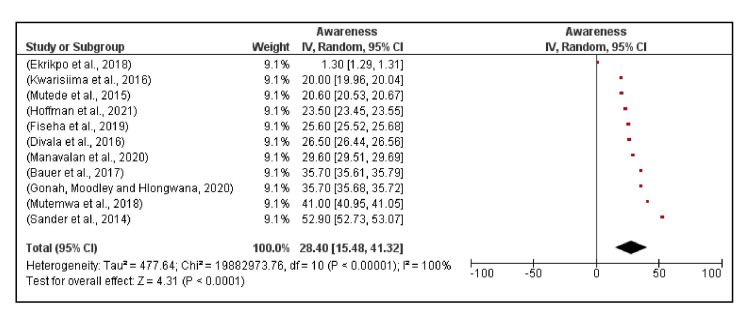
Awareness of hypertension among PLHIV across 11 studies in Sub-Saharan Africa. Persons living with human immunodeficiency virus (PLHIV) [29, 35, 39-43, 50, 52-53]

The awareness of hypertension among hypertensive PLHIV in SSA ranged from 1.3% in the Nigerian study to 52.9% in the study from Uganda [40, 53]. The median awareness was 26.5%, and the random effects model pooled awareness was 28.4% (95% CI, 15.5%, 41.3%) (Figure 5).

**Figure 6:**
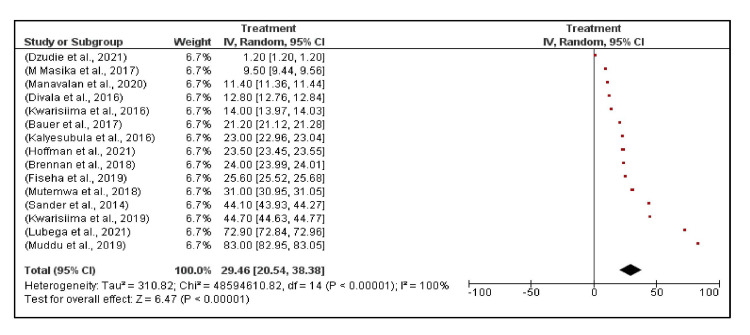
Treatment of hypertension among PLHIV across 15 studies in Sub-Saharan Africa. Persons living with human immunodeficiency virus (PLHIV) [10, 15, 29, 35, 37, 39, 41, 43-44, 46-47]

The level of hypertension treatment initiation among PLHIV with hypertension in SSA ranged from 1.2% (Dzudie et al., Cameroon) [10] to 83.0% (Muddu et al., Uganda) [15]. The median hypertension treatment initiation was 23.5%, and the random effects model pooled treatment initiation was 29.5% (95% CI, 20.5%, 38.4%) (Figure 6).

**Figure 7:**
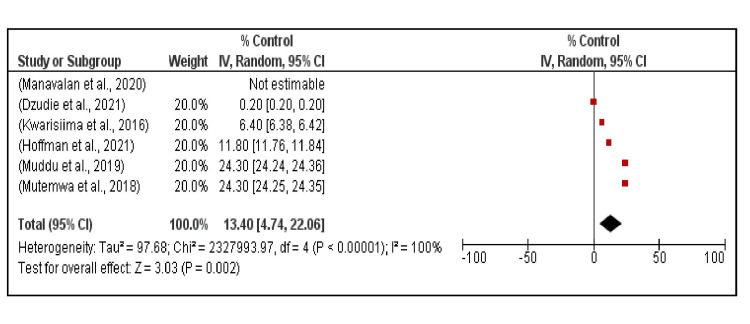
Hypertension control among PLHIV across six studies in Sub-Saharan Africa. Persons living with human immunodeficiency virus (PLHIV) [10, 15, 29, 43, 46, 50]

Hypertension control among PLHIV with hypertension in SSA ranged from 0% (Manavalan et al., Tanzania) [50] to 24.3% (Hoffman et al., Kwarisiima et al., Malawi and Uganda respectively) [43, 46]. The median hypertension control was 9.1%, and the random effects model pooled hypertension control was 13.4% (95% CI, 4.7%, 22.1%) (Figure 7).

Discussion

In this review, we found a high prevalence of hypertension among PLHIV in SSA, with low levels of hypertension awareness, treatment, and control. The pooled prevalence of hypertension among PLHIV in SSA of 19.6% was lower than the estimated hypertension prevalence in the general SSA population of 30% [25] or the global estimates of 25.2% [58]. The relatively lower prevalence of hypertension observed among PLHIV may be attributed to the routine lifestyle counselling offered in most HIV clinics that incorporates health education on modifiable risk factors for CVD like, healthy diet, physical exercise, weight control and low salt intake, which may confer benefit on hypertension prevalence [55]. In addition, immune suppression, HIV wasting syndrome and malnutrition, which are not uncommon among PLHIV in SSA, are associated with lower prevalent hypertension [36].

The WHO’s HIV guidelines recommend routine screening of all adult PLHIV for hypertension on every clinic visit [12], however, this was inconsistently implemented across most HIV clinics. Only a few studies reported routine screening of hypertension among PLHIV [35, 45, 53]. In some clinics, blood pressure measurements were done by a clinician at their discretion, usually when a patient presented with signs and symptoms of hypertension, and only 27.7% of all eligible PLHIV were screened within one year of enrolment into HIV care [15]. Indiscriminate screening of hypertension in all adults is recommended for early hypertension diagnosis and management.

We found a low hypertension awareness of 26.5% ranging from 1.3% by Ekrikpo et al. [40] to 52.9% by Sander et al. [53]. This low hypertension awareness was similar to the 27% found in the general SSA population [25], indicating existing gaps in hypertension control programs in SSA. The low hypertension awareness in community surveys in SSA has been attributed to infrequent contact of individuals with health systems [25]. However, a low hypertension awareness among PLHIV indicates a missed opportunity to screen and diagnose hypertension, as PLHIV interact more often with the health system compared to the general population.

There was inconsistent reporting on hypertension treatment by most studies, which could be a lack of these data elements in the HIV patient records. We observed hypertension treatment initiation level of 29.5% of those diagnosed with hypertension, which was slightly higher than the 18% observed in the general population of SSA [25]. Even in settings where hypertension medicines are generally available in government facilities like in South Africa, Mutemwa et al. reported that there were still low levels of hypertension treatment initiation at 31% [29]. This was attributed to structural barriers like unavailability of hypertension medicines in the HIV clinic pharmacy and a lack of clear guidance for hypertension diagnosis, treatment initiation and monitoring among PLHIV. Compared to medicines for infectious diseases like malaria and tuberculosis, there was low access to NCDs medicines at about 44% for health facilities in SSA [59]. Availability of NCDs medicines within HIV facilities can overcome these structural barriers, limit duplication of services, and thus save patients’ time and transport costs [60].

From this review, the commonly prescribed hypertension medicines in SSA include, Nifedipine, Atenolol, Propranolol, Furosemide, and thiazide diuretics (Table 5). These medicines are preferentially procured by government facilities due to their relatively low cost. However, they are often associated with sub-optimal blood pressure control, undesirable side effects and often require multiple daily dosing that affects compliance and adherence to these medicines. Patients usually resort to buying alternative anti-hypertensive medicines out-of-pocket from private pharmacies or resolve not to take any medicines altogether due to the asymptomatic nature of hypertension [60].

Overall, we observed sub-optimal hypertension control of 13.4% ranging from 0% by Manavalan et al. [50] to 24.3% by Hoffman et al. [43]. The major barriers to optimal hypertension control were poor access to anti-hypertensive medicines and frequent drug stock outs [10, 29, 42]. Notably, frequent clinic visits not clinically indicated but due to shorter drug refills were a negative predictor of hypertension control in patients receiving integrated hypertension and HIV care [9]. Alternatively, studies have illustrated the feasibility of integrated multi-month dispensing of both hypertension and HIV drug refill for stable PLHIV for dual control [61].

The high prevalence of hypertension observed in this review was predominantly driven by traditional risk factors such as age, overweight, obesity and level of physical exercises, and to a lesser extent mediated by HIV-related factors such as ART use, duration on ART, immune status and nadir CD4 count [10, 62]. However, HIV viral suppression did not predict hypertension [46]. But increasing age, overweight, and obesity were consistently associated with hypertension. Advanced age is a known risk factor for hypertension, but the onset of hypertension in PLHIV seems to occur earlier in life compared to HIV negative counterparts [54]. Among PLHIV, BMI was a better predictor of hypertension in men than age, while age was a better predictor of hypertension in women than BMI [36], but this gender predilection was consistently not observed in most studies. PLHIV with multiple comorbidity would require all-round indicators that inform and monitor their care beyond HIV related parameters for overall improved quality of life [44].

Further research is needed to understand the optimal and context appropriate HIV and hypertension integration models that are designed to reduce patient level barriers, efficient and optimize health systems for chronic care delivery. There is limited knowledge about the health systems factors that might influence hypertension care while leveraging HIV infrastructure for persons with or without HIV infection. Like how clinic appointment schedules, waiting time, patient flow and stigma may affect HIV and hypertension integration models [9]. As HIV programs in SSA are moving towards attaining the Joint United Nations Programme on HIV/AIDS (UNAIDS) 95-95-95 goals of diagnosis, treatment initiation and HIV viral suppression, care for highly prevalent comorbid condition like hypertension -- a major CVD risk factor -- seems to lag behind. If left unchecked, adverse hypertension-related morbidity and mortality will undermine the gains of the HIV program in SSA. This underscores the need for differentiated services that are tailored to PLHIV with hypertension.

Study limitations

Most studies that met the inclusion criteria were clinic-based with potential selection bias for patients who were able to come to the clinics. This could have led to an estimate that is not representative of the general community. However, most SSA countries have more than 70% of PLHIV enrolled in care, and the clinic prevalence of hypertension among PLHIV may be a close estimate of the true prevalence. Also, number of studies included in the review reported poor documentation of blood pressure measurements and the prescribed antihypertensive medication in the patients’ files, which could have led to under estimating the hypertension prevalence and treatment initiation. All studies utilized clinic-based (office) blood pressure measurements that could misclassify individuals with white coat or masked hypertension whose prevalence in SSA is estimated to be 15% and 11%, respectively [63]. However, these misclassifications would likely indicate a higher prevalence than estimated in this review.

## Conclusions

There is a large burden of hypertension among PLHIV in SSA that is largely under diagnosed and sub-optimally controlled. We recommend strengthening hypertension screening and control programs through routine screening all PLHIV for hypertension, improving access to hypertension medicines and adopting models of integrating HIV and hypertension services. The high prevalence of hypertension observed in this review was predominantly driven by traditional risk factors such as age, overweight, obesity and level of physical exercises, and to a lesser extent mediated by HIV-related factors such as ART use, duration on ART, immune status, and nadir CD4 count. The HIV viral suppression did not predict hypertension, however increasing age, overweight, and obesity were consistently associated with hypertension. Among persons living with HIV, BMI was a better predictor of hypertension in men than age, while age was a better predictor of hypertension in women than BMI but this gender predilection was consistently not observed in most studies.

## Appendices

**Table 5:** Key terms used in the literature search.

Category	Search term
#1	Prevalence OR Epidemiology OR Awareness
#2	Hypertension OR Blood pressure OR BP
#3	HIV OR Human Immunodeficiency Virus OR AIDS
#4	"Africa South of the Sahara" OR Sub-Saharan Africa OR "Sub Saharan Africa"
#5	Angola OR Benin OR Botswana OR "Burkina Faso" OR "Upper Volta" OR Burundi OR Urundi OR Cameroon OR Cameroons OR "Cape Verde" OR "Central African Republic" OR Chad OR Comoros OR "Comoro Islands" OR Comores OR Mayotte OR Congo OR Zaire OR "Cote d'Ivoire" OR "Ivory Coast" OR "Democratic Republic of the Congo" OR Djibouti OR "French Somaliland" OR Eritrea OR Ethiopia OR Gabon OR "Gabonese Republic" OR Gambia OR Ghana OR "Gold Coast" OR Guinea OR Kenya OR Lesotho OR Basutoland OR Liberia OR Madagascar OR "Malagasy Republic" OR Malawi OR Nyasaland OR Mali OR Mauritania OR Mauritius OR Mozambique OR Namibia OR Niger OR Nigeria OR Rwanda OR "Sao Tome" OR Seychelles OR Senegal OR "Sierra Leone" OR Somalia OR "South Africa" OR Sudan OR Swaziland OR Tanzania OR Togo OR "Togolese Republic" OR Uganda OR Zambia OR Zimbabwe OR Rhodesia
#6	#4 OR #5
#7	#1 AND #2 AND #3 AND #6
	Filters: Publication date from 2000/01/01 to 2022/03/31; Humans

**Table 6:** Risk of bias assessment scores based on the NIH quality assessment tool for observational cohort and cross-sectional studies. NIH, National Institutes of Health

	Sander et al. [53]	Bloomfield et al. [36]	Divala et al. [39]	Kalyesubula et al. [44]	Kwarisiima et al. [46]	Bauer et al. [35]	Manne-Goehler et al. [54]	Masika et al. [49]	Brennan et al. [37]	Ekrikpo et al. [40]	Mutemwa et al. [29]	Fiseha et al. [41]	Kansiime et al. [45]	Kwarisiima et al. [46]	Muddu et al. [15]	Mutede et al. [52]	Gonah et al. [42]	Manavalan et al. [50]	Mathebula et al. [51]	Chiwandire et al. [38]	Dzudie et al. [10]	Hoffman et al. [43]	Lubega et al. [47]	Lukas et al. [48]
Was the research question or objective in this paper clearly stated?	Yes	Yes	Yes	Yes	Yes	Yes	Yes	Yes	Yes	Yes	Yes	Yes	Yes	Yes	Yes	Yes	Yes	Yes	Yes	Yes	Yes	Yes	Yes	Yes
Was the study population clearly specified and defined?	Yes	Yes	Yes	Yes	Yes	Yes	Yes	Yes	Yes	Yes	Yes	Yes	Yes	Yes	Yes	Yes	Yes	Yes	Yes	Yes	Yes	Yes	Yes	Yes
Was the participation rate of eligible persons at least 50%?	Yes	No	Yes	Yes	Yes	Yes	Yes	Yes	Yes	No	Yes	Yes	Yes	Yes	No	Yes	Yes	Yes	Yes	Yes	Yes	Yes	Yes	Yes
Were all the subjects selected or recruited from the same or similar populations (the same time period)?	Yes	Yes	Yes	Yes	Yes	Yes	No	Yes	Yes	Yes	Yes	Yes	Yes	Yes	Yes	Yes	Yes	Yes	Yes	Yes	Yes	Yes	Yes	Yes
Were inclusion and exclusion criteria for being in the study pre-specified and applied uniformly to all participants?	Yes	Yes	Yes	Yes	Yes	No	No	Yes	Yes	Yes	Yes	Yes	Yes	Yes	Yes	Yes	Yes	Yes	Yes	Yes	Yes	Yes	Yes	Yes
Was a sample size justification, power description, or variance and effect estimates provided?	No	No	Yes	Yes	Yes	No	Yes	No	No	No	Yes	Yes	Yes	No	No	Yes	Yes	No	No	No	No	No	No	Yes
For the analyses in this paper, were the exposure(s) of interest measured prior to the outcome(s) being measured?	Yes	Yes	Yes	Yes	Yes	Yes	Yes	Yes	Yes	Yes	Yes	Yes	Yes	Yes	Yes	Yes	Yes	Yes	Yes	Yes	Yes	Yes	Yes	Yes
Was the timeframe sufficient so that one could reasonably expect to see an association between exposure and outcome if it existed?	Yes	Yes	No	Yes	Yes	Yes	Yes	Yes	No	No	Yes	No	No	Yes	Yes	Yes	Yes	Yes	Yes	Yes	Yes	Yes	Yes	Yes
Were the exposure measures (independent variables) clearly defined, valid, reliable, and implemented consistently across all study participants?	Yes	Yes	Yes	Yes	Yes	Yes	Yes	Yes	Yes	Yes	Yes	Yes	Yes	Yes	Yes	Yes	Yes	Yes	Yes	Yes	Yes	Yes	Yes	Yes
Was the exposure(s) assessed more than once over time?	Yes	Yes	Yes	Yes	Yes	Yes	Yes	Yes	Yes	No	Yes	Yes	No	Yes	Yes	No	Yes	Yes	Yes	No	No	Yes	Yes	Yes
Were the outcome measures (dependent variables) clearly defined, valid, reliable, and implemented consistently across all study participants?	Yes	Yes	Yes	Yes	Yes	Yes	Yes	Yes	Yes	Yes	Yes	Yes	Yes	Yes	Yes	Yes	Yes	Yes	Yes	Yes	Yes	Yes	Yes	Yes
Were the outcome assessors blinded to the exposure status of participants?	No	No	No	No	No	No	No	No	No	No	No	No	No	No	No	No	No	No	No	No	No	No	No	No
Was loss to follow-up after baseline ≤ 20%?	Yes	No	Yes	Yes	Yes	Yes	No	Yes	Yes	Yes	Yes	Yes	Yes	Yes	Yes	Yes	Yes	Yes	Yes	Yes	Yes	Yes	Yes	Yes
Were key potential confounding variables measured and adjusted statistically for their impact on the relationship between exposure(s) and outcome(s)?	Yes	Yes	Yes	Yes	Yes	Yes	Yes	Yes	Yes	Yes	Yes	Yes	No	Yes	Yes	Yes	Yes	No	Yes	Yes	Yes	Yes	Yes	Yes
Total Score (Yes) out of 14	12	10	12	13	13	11	10	12	11	9	13	12	10	12	11	12	13	11	12	11	11	12	12	13
